# Bitter-RF: A random forest machine model for recognizing bitter peptides

**DOI:** 10.3389/fmed.2023.1052923

**Published:** 2023-01-26

**Authors:** Yu-Fei Zhang, Yu-Hao Wang, Zhi-Feng Gu, Xian-Run Pan, Jian Li, Hui Ding, Yang Zhang, Ke-Jun Deng

**Affiliations:** ^1^School of Life Science and Technology, Center for Informational Biology, University of Electronic Science and Technology of China, Chengdu, China; ^2^Innovative Institute of Chinese Medicine and Pharmacy, Academy for Interdiscipline, Chengdu University of Traditional Chinese Medicine, Chengdu, China; ^3^School of Basic Medical Sciences, Chengdu University, Chengdu, China

**Keywords:** bitter peptide, sequence information, random forest, feature fusion, classification method

## Abstract

**Introduction:**

Bitter peptides are short peptides with potential medical applications. The huge potential behind its bitter taste remains to be tapped. To better explore the value of bitter peptides in practice, we need a more effective classification method for identifying bitter peptides.

**Methods:**

In this study, we developed a Random forest (RF)-based model, called Bitter-RF, using sequence information of the bitter peptide. Bitter-RF covers more comprehensive and extensive information by integrating 10 features extracted from the bitter peptides and achieves better results than the latest generation model on independent validation set.

**Results:**

The proposed model can improve the accurate classification of bitter peptides (AUROC = 0.98 on independent set test) and enrich the practical application of RF method in protein classification tasks which has not been used to build a prediction model for bitter peptides.

**Discussion:**

We hope the Bitter-RF could provide more conveniences to scholars for bitter peptide research.

## 1. Introduction

The bitter peptides, often produced in fermented, aged, and spoiled foods, are oligopeptides with diverse structures. Studies have shown that hydrophobic amino acids and their positions are crucial determinants for bitter peptides to exhibit bitter taste (1, 2). Experiments have found that many toxins are bitter taste, so most mammals, including humans, avoid the intake of toxins by avoiding bitter substances (3). However, some bitter substances may have medicinal effects. In biomedical and clinical sciences, hormetic responses were of considerable importance. Many drugs displayed hormetic-like biphasic dose responses and showed opposite effects at low and high doses (4). In diabetic patients, the peptides in *Momordica charantia* (*M. charantia*) can significantly regulate blood glucose concentration. A 68-residue insulin receptor binding protein was isolated from *M. charantia*. MclRBP-19 in this protein can span the 50th-68th residues, enhance the binding of insulin and IR, stimulate the phosphorylation of PDK1 and Akt, and induce the expression of glucose transporter 4, thus promoting glucose clearance (5). And frequent consumption of *M. charantia* peptide is beneficial to multiple organs of human body (6). The active compound polypeptide K extracted from the seeds of *M. charantia* has gastroprotective effects in some gastric ulcer models (7). Hence, bitter peptides, previously avoided due to their potential toxicity, can be beneficial at the correct dosage. Consequently, the bitter peptides may be very useful in medicine, making their identification extremely important (8).

Experimental methods for identifying bitter peptides have a solid theoretical basis, but the operation is complex, time-consuming, and inaccurate. Biological methods often involve the extraction of bitter peptides from raw materials through gel separation, multiple rounds of liquid chromatography separation, and purification. Finally, Fourier transforms infrared spectroscopy (FTIR) was used to identify bitter peptides. Generally, spectroscopic-based methods have requirements for instruments, which are not universal (9, 10). Therefore, the bitterness evaluation stage requires the participation of human subjects, which may lead to inaccurate results (11, 12). Bioinformatics-based methods for predicting bitter peptides have the advantages of no professional instrument requirements, short time consumption, and high prediction accuracy. Therefore, it is imperative to develop a machine learning model for predicting bitter peptides.

At present, computational methods have been carried out to study peptides (13, 14). Models based on the quantitative structure of bitter taste relationship (QSBR), including multiple linear regression, the support vector machine (SVM), and artificial neural network (ANN), have been used to predict bitter peptides (2, 15–21). Specifically, based on 229 experimental bitterness values determined by human sensory evaluations, Dragon 5.4 software was designed to predict bitter peptides by extracting 1292 descriptors and reducing descriptors to 244 using a home-developed toolbox. Then, the GA-PLS method was used to select the six best-scoring descriptors for the QSAR model construction. The six descriptors, including SPAN, Mean square distance (MSD), E3s, G3p, Hats8U, and 3D-MoRSE, represent the dimension of the molecule, the numbers of atoms, weighted atomic electrical topological states, the 3rd-component symmetry directional WHIM index (weighed by polarizability), spatial autocorrelation-based descriptors and an indicator of size, mass, and volume of the molecules.

Further, to improve prediction accuracy, four generations of classification models based on bitter peptide sequences have been developed. The first-generation model used dipeptide propensity scores to predict bitter peptides by extracting a few characteristics of bitter peptides (22). The second-generation model utilized deep learning research methods. However, there may be problems with information redundancy and overfitting (23). The third-generation model integrated five peptide features to formulate bitter peptides, but the representativeness should be further optimized (24, 25). The fourth-generation model extracted feature extraction by deep learning pre-training, and then built a prediction model based on light gradient boosting machine (LGBM) (26).

**FIGURE 1:**
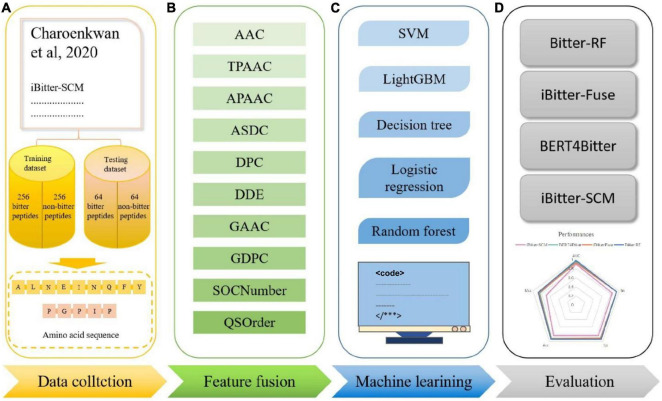
Schematic framework of the Bitter peptide prediction model (Bitter–RF). The main process of Bitter-RF design mainly includes the following steps: **(A)** dataset collection, **(B)** feature fusion, **(C)** modeling with multiple machine learning methods, **(D)** Bitter-RF performance evaluation.

Inspired by the previous four generations of models, we proposed Bitter-RF, a novel machine learning method for predicting bitter peptides. In total, ten kinds of feature information were extracted, consisting of 1,337 features in the feature set. By deleting all zero items, 1206 features were used for model learning. Here, we used five machine learning models to learn the features. After comparison, the RF method has the best classification effect. The schematic framework of Bitter-RF for bitter peptide prediction is shown in Figure 1.

## 2. Materials and methods

### 2.1. Dataset source

The fundamental for constructing a powerful model is to generate a high-quality benchmark dataset. To provide a reliable model and make a fair comparison, we used the same dataset as the previous four generation models (22–24), which can be obtained from http://pmlab.pythonanywhere.com/BERT4Bitter (accessed on 13 January 2022). This data was originally obtained by manually collecting experimentally validated bitter peptides from various literatures (22). The data contains 640 records, including 320 experimentally validated bitter peptides and 320 non-bitter peptides, which were randomly generated from BIOPEP. In order to objectively evaluate the model, we divided the data into training set and independent set at a ratio of 8:2. The training set contains 256 bitter peptides and 256 non-bitter peptides. The independent set contains 64 bitter peptides and 64 non-bitter peptides.

### 2.2. Feature extraction

In a computational model based on machine learning methods for biological sequence data, the coding methods of sequences, which can reveal as much sequence information as possible, are the most critical step (27–36). In the field of sequence analysis, scholars have done a lot of works, and various of sequence descriptors were proposed. Here, we used iLearnPlus to extract 10 types of features of bitter peptides (37). The specific information was described as follows.

### 2.2.1. Amino acid composition (AAC)

The AAC encoding calculates the frequencies of 20 natural amino acids in a peptide sequence (38–42). The equation was shown as follows.


(1) $$f\,\left(t\right)=\frac{N\,\left(t\right)}{N},t\in \left\{A,C,\ldots ,Y\right\}$$

where *N*(*t*) means the number of amino acid type *t*, and *N* means the length of peptides.

### 2.2.2. Traditional pseudo-amino acid composition (TPAAC)

The TPAAC descriptor is proposed by Chou, which is also called the type1 pseudo-amino acid composition (43). Here, we use$H_{1}^{0}\,\left(i\right)$, $H_{2}^{0}\,\left(i\right)$, and *M*^0^(*i*) (i = 1, 2, 3,, 20) to respectively represent the original hydrophobicity values (44), original hydrophilicity values (45) and original side chain masses of 20 natural amino acids. We normalized these values based on the standard normal distribution, as follows.


(2) $$H_{1}\,\left(i\right)=\frac{H_{1}^{o}\,\left(i\right)-\frac{1}{20}\,\sum_{i=1}^{20}H_{1}^{o}\,\left(i\right)}{\sqrt{\frac{\sum_{i=1}^{20}{\left[H_{1}^{o}\,\left(i\right)-\frac{1}{20}\,\sum_{i=1}^{20}H_{1}^{o}\,\left(i\right)\right]}^{2}}{20}}}$$

(3) $$H_{2}\,\left(i\right)=\frac{H_{2}^{o}\,\left(i\right)-\frac{1}{20}\,\sum_{i=1}^{20}H_{2}^{o}\,\left(i\right)}{\sqrt{\frac{\sum_{i=1}^{20}{\left[H_{2}^{o}\,\left(i\right)-\frac{1}{20}\,\sum_{i=1}^{20}H_{2}^{o}\,\left(i\right)\right]}^{2}}{20}}}$$

(4) $$M\,\left(i\right)=\frac{M^{0}\,\left(i\right)-\frac{1}{20}\,\sum_{i=1}^{20}M^{0}\,\left(i\right)}{\sqrt{\frac{\sum_{i=1}^{20}{\left[M^{0}\,\left(i\right)-\frac{1}{20}\,\sum_{i=1}^{20}M^{0}\,\left(i\right)\right]}^{2}}{20}}}$$

Then, the correlation function for residues *R*_*i*_ and *R*_*j*_ can be defined as:


(5) $$\Theta \left(R_{i},R_{j}\right)=\frac{1}{3}\{{\left[H_{1}\left(R_{i}\right)-H_{1}\left(R_{j}\right)\right]}^{2}+{\left[H_{2}\left(R_{i}\right)-H_{2}\left(R_{j}\right)\right]}^{2}$$

$$+{\left[M\left(R_{i}\right)-M\left(R_{j}\right)\right]}^{2}\}$$

The correlation function contains the three amino acid properties mentioned above. By generalizing this function definition, an amino acid property (Eq. 6) and a set of amino acid properties (Eq.7) are defined.


(6) $$\Theta \,\left(R_{i},R_{j}\right)={\left[H_{1}\,\left(R_{i}\right)-H_{1}\,\left(R_{j}\right)\right]}^{2}$$

(7) $$\Theta \,\left(R_{i},R_{j}\right)=\frac{1}{n}\,\sum_{n=1}^{n}{\left[H_{k}\,\left(R_{i}\right)-H_{k}\,\left(R_{j}\right)\right]}^{2}$$

where *H* (*R*_*i*_) is the amino acid property of amino acid *R*_*i*_ after standardization and *H*_*k*_ (*R*_*i*_) is the *k*-th attribute in the amino acid attribute set of amino acid *R_i_*. And sequence order-correlated factors were defined as:


(8) $$\theta_{1}=\frac{1}{N-1}\,\sum_{i=1}^{N-1}\Theta \,\left(R_{i},R_{i+1}\right)$$

(9) $$\theta_{2}=\frac{1}{N-2}\,\sum_{i=1}^{N-2}\Theta \,\left(R_{i},R_{i+2}\right)$$

…


(10) $$\theta_{\lambda }=\frac{1}{N-\lambda }\,\sum_{i=1}^{N-\lambda }\Theta \,\left(R_{i},R_{i+\lambda }\right)$$

where λ is a correlation parameter that can be adjusted, and λ should be less than *N*, we set λ = 1. And traditional pseudo-amino acid composition for a protein sequence can be defines as:


(11) $$X_{c}=\frac{f_{c}}{\sum_{r=1}^{20}f_{r}+\omega \,\sum_{j=1}^{\lambda }\theta_{j}},\left(1<c<20\right)$$

(12) $$X_{c}=\frac{\omega \,\theta_{c-20}}{\sum_{r=1}^{20}f_{r}+\omega \,\sum_{j=1}^{\lambda }\theta_{j}},\left(21<c<20+\lambda \right)$$

where ω is the weigthing factor and is set to 0.05 in this study.

### 2.2.3. Amphiphilic pseudo-amino acid composition (APAAC)

The APAAC is a kind of PseAAC. It contains 20+2λ discrete numbers: the first 20 numbers consist of conventional amino acids; the next 2λ numbers are a set of correlation factors that reflect different distribution patterns of hydrophobicity and hydrophilicity along the peptide chain (46). This feature was described as follows.

Firstly, using *H*_1_(*i*)(Eq.2) and *H*_2_(*i*)(Eq.3) which are defined in TPAAC to define hydrophobicity and hydrophilicity correlation functions:


(13) $$H_{i,j}^{1}=H_{1}\,\left(i\right)\,H_{1}\,\left(j\right)$$

(14) $$H_{i,j}^{2}=H_{2}\,\left(i\right)\,H_{2}\,\left(j\right)$$

Secondly, sequence order factors can be formulated as:


(15) $$\tau_{1}=\frac{1}{N-1}\,\sum_{i=1}^{N-1}H_{i,i+1}^{1}$$

(16) $$\tau_{2}=\frac{1}{N-1}\,\sum_{i=1}^{N-1}H_{i,i+1}^{2}$$

(17) $$\tau_{3}=\frac{1}{N-2}\,\sum_{i=1}^{N-2}H_{i,i+2}^{1}$$

(18) $$\tau_{4}=\frac{1}{N-2}\,\sum_{i=1}^{N-2}H_{i,i+2}^{2}$$

…


(19) $$\tau_{2\,\alpha -1}=\frac{1}{N-\alpha }\,\sum_{i=1}^{N-\alpha }H_{i,i+\alpha }^{1}$$

(20) $$\tau_{2\,\alpha }=\frac{1}{N-\alpha }\,\sum_{i=1}^{N-\alpha }H_{i,i+\alpha }^{2}$$

Finally, the APAAC is defined as:


(21) $$P_{C}=\frac{f_{c}}{\sum_{r=1}^{20}f_{r}+w\,\sum_{j=1}^{2\,\lambda }\tau_{j}},\left(1<c<20\right)$$

(22) $$P_{C}=\frac{\omega \,\tau_{u}}{\sum_{r=1}^{20}f_{r}+w\,\sum_{j=1}^{2\,\lambda }\tau_{j}},\left(21<u<20+2\lambda \right)$$

where w is the weighting factor, and it is set to 0.5 in this study. This value refers to Chou’s work on protein cell property prediction using this feature (43). And we set λ 1 in this study.

### 2.2.4. Adaptive skip dinucleotide composition (ASDC)

ASDC is a modified dipeptide composition, which takes full account of the relevant information that exists between adjacent residues and between intervening residues. The feature vector for ASDC was defined as:


$$\text{ASDC}=\left(f_{v\,1},f_{v\,2},\ldots ,f_{v\,400}\right),$$

(23) $$f_{v\,i}=\frac{\sum_{g=1}^{L-1}O_{i}^{g}}{\sum_{i=1}^{400}\sum_{g=1}^{L-1}O_{i}^{g}}$$

where *f*_*vi*_ means the occurrence frequency of all possible dipeptide with ≤ *L-1* intervening peptides.

### 2.2.5. Di-peptide composition (DPC)

The DPC encoding describes the frequencies of 400 dipeptide combination in peptide sequence (47). The calculation method was shown as follows.


(24) $$D\,\left(r,s\right)=\frac{N_{r\,s}}{N-1},r,s\in \left\{A,C,D,\ldots ,Y\right\}$$

where *N*_*rs*_ means the number of dipeptides combined by amino acid types *r* and amino acid types *s* and *N* is the length of peptide.

### 2.2.6. Dipeptide deviation from expected mean (DDE)

DDE includes three parameters: dipeptides composition (*D_c_*), theoretical mean (*T_m_*), and theoretical variance (*T_v_*). *D_c_* is the same as DPC’s calculation method.*T_m_* and *T_v_* were calculated as follows:


(25) $$T_{m}\,\left(r,s\right)=\frac{C_{r}}{C_{N}}\times \frac{C_{s}}{C_{N}}$$

(26) $$T_{v}\,\left(r,s\right)=\frac{T_{m}\,\left(r,s\right)\,\left(1-T_{m}\,\left(r,s\right)\right)}{N-1}$$

where *C_r_* means the number of codons for the amino acid types *r*, and *C_s_* means the number of codons for the amino acid types *s*. *C_N_* includes total possible codons, which means not including the three stop codons.

Using three parameters, DDE was calculated as follows:


(27) $$D\,D\,E\,\left(r,s\right)=\frac{D_{c}\,\left(r,s\right)-T_{m}\,\left(r,s\right)}{T_{v}\,\left(r,s\right)}$$

### 2.2.7. Grouped amino acid composition (GAAC)

GAAC divides 20 amino acids into five groups based on their physicochemical properties that are the aliphatic group (*g1*: GAVLMI), aromatic group (*g2*: FYW), positive charge group (*g3*: KRH), negative charged group (*g4*: DE) and uncharged group (*g5*: STCPNQ). This feature describes the frequencies of these five groups of amino acids and can be calculated as follows:


(28) $$f\,\left(g\right)=\frac{N\,\left(g\right)}{N},G\in \left\{g\,1,g\,2,g\,3,g\,4,g\,5\right\}$$

where *N*(*g*) is the sum of the number of the amino acid which belongs to group *g*, and *N* is the length of peptide sequence.

### 2.2.8. Grouped dipeptide composition (GDPC)

GDPC is a variant of DPC based on the amino acid classification already mentioned in GAAC. The feature consists of 25 descriptors, calculated as follows:


(29) $$f\,\left(r,s\right)=\frac{N_{r\,s}}{N-1},r,s\in \left\{g\,1,g\,2,g\,3,g\,4,g\,5\right\}$$

where *N*_*rs*_ is the number of dipeptides represented by amino acid type groups r and s, and *N* is the length of peptide sequence.

### 2.2.9. Sequence-order-coupling number (SOCNumber)

The *d*-th rank sequence-order-coupling number was calculated as follows:


(30) $$\tau_{d}=\sum_{i=1}^{N-d}{\left(d_{i,i+d}\right)}^{2},d=1,2,\ldots ,n\,l\,a\,g$$

where *d*_*i,i+d*_ describes the distance between two amino acids at positions *i* and *i + d* in a given distance matrix, *nlag* denotes the maximum value of the lag (default value: 30) and *N* is the length of the peptide sequence. The distance matrix used here from both Schneider–Wrede physicochemical distance matrix (48) and Grantham chemical distance matrix (49).

### 2.2.10. Quasi-sequence-order (QSOrder)

For each amino acid, defined QSOrder as follows:


(31) $$X_{r}=\frac{f_{r}}{\sum_{r=1}^{20}f_{r}+w\,\sum_{d=1}^{n\,l\,a\,g}\tau_{d}},r=1,2,3,\ldots ,20$$

where *f_r_* represent the normalized occurrence of amino acid which is *r* typed, and the weighting factor *w* is defined as 0.1, and *nlag* denotes the maximum value of the lag (default value: 30). τ_*d*_ is the same as the definition in SOCNumber.

For other 30 quasi-sequence-order descriptors, defined QSOrder as follows:


(32) $$X_{d}=\frac{w\,\tau_{d}-20}{\sum_{r=1}^{20}f_{r}+w\,\sum_{d=1}^{n\,l\,a\,g}\tau_{d}},d=21,22,\ldots ,20+n\,l\,a\,g$$

### 2.3. Random forest

RF algorithm is an ensemble of decision trees and has been widely used for classification. Each tree depends on the value of a random vector that is sampled independently and has the same distribution for all trees in the forest. The introduction of randomness can reduce the possibility of overfitting, improve the ability to resist noise, and has strong adaptability to high-dimensional data.

RF algorithm has been applied to a variety of protein classification problems (50–54).

### 2.4. Model evaluation metrics

To evaluate the training effect and prediction ability of the model, we mainly used the Area Under the Receiver Operating Characteristic curve value (AUROC), supplemented by Sensitivity (Sn), Specificity (Sp), Matthew’s correlation coefficient (MCC), accuracy (ACC) (55–72). These indexes can be formulated as follows:


(33) $$S\,n=\frac{T\,P}{\left(T\,P+F\,N\right)}$$

(34) $$S\,p=\frac{T\,N}{\left(T\,N+F\,P\right)}$$

(35) $$M\,C\,C=\frac{\left(T\,N\times T\,P-F\,N\times F\,P\right)}{\sqrt{\left(T\,P+F\,P\right)\,\left(T\,P+F\,N\right)\,\left(T\,N+F\,P\right)\,\left(T\,N+F\,N\right)}}$$

(36) $$A\,C\,C=\frac{\left(T\,P+T\,N\right)}{\left(T\,P+T\,N+F\,P+F\,N\right)}$$

where *TP* and *FN* represent the number that the bitter peptides are predicted as true bitter peptides and non-bitter peptides, respectively. On the contrary, *TN* and *FP* represent the number that the non-bitter peptides are predicted as true non-bitter peptides and bitter peptides, respectively. That is to say, bitter peptides were defined as positive samples, and non-bitter peptides were defined as negative samples in this work.

Sn is the model’s sensitivity, representing the proportion of correctly predicted positive samples to the total number of actual positive samples (73–76). Sp is the model’s specificity, representing the proportion of correctly predicted negative samples to the total number of actual negative samples (77, 78). Here ACC, MCC and AUROC are all comprehensive indicators. ACC represents the proportion of correct predicted samples to the total samples. And MCC is the correlation coefficient between the description classification and the predicted classification. Its range is [-1, 1]. If the value is 1, it means the model prediction performance is perfect. If the value is -1, it means the prediction is completely opposite to the actual. The AUROC indicator can be used as a standard for evaluating the quality of the binary classification model (79–82). The closer the value of AUROC is to 1, the better the classification effect.

## 3. Results and discussion

### 3.1. Single-feature-based results

Here, we used iLearnPlus to extract the above 10 features (AAC, TPAAC, APAAC, ASDC, DPC, DDE, GAAC, GDPC, SOCNumber, QSOrder) and then utilized them to train a RF-based predictive model for accurately identifying Bitter peptides (37). Table 1 shows the results of 10-fold cross-validation and independent set.

**TABLE 1:** Results of RF-based models using 10 single features.

Cross-validation	Feature	Dimension	AUROC	Sn	Sp	Acc	Mcc
10-fold cross-validation	AAC	20	**0.91**	0.85	**0.84**	**0.85**	**0.69**
	TPAAC	21	0.90	0.83	0.78	0.80	0.61
	APAAC	22	0.89	0.83	0.81	0.82	0.64
	ASDC	400	0.88	**0.89**	0.68	0.79	0.59
	DPC	400	0.86	0.87	0.64	0.76	0.53
	DDE	400	0.83	0.84	0.73	0.78	0.57
	GAAC	5	0.75	0.72	0.66	0.69	0.39
	GDPC	25	0.78	0.75	0.71	0.73	0.46
	SOCNumber	2	0.70	0.66	0.62	0.64	0.28
	QSOrder	42	0.89	0.82	0.82	0.82	0.64
Independent set validation	AAC	20	0.96	0.91	0.89	**0.90**	**0.80**
	TPAAC	21	0.94	0.83	0.86	0.84	0.69
	APAAC	22	**0.97**	0.89	**0.91**	**0.90**	0.80
	ASDC	400	0.92	0.89	0.75	0.82	0.65
	CKSAAGP	100	0.87	0.77	0.81	0.79	0.58
	DPC	400	0.89	0.88	0.70	0.79	0.59
	DDE	400	0.90	0.89	0.84	0.87	0.74
	GAAC	5	0.76	0.83	0.64	0.73	0.48
	GDPC	25	0.80	0.73	0.72	0.73	0.45
	SOCNumber	2	0.73	0.59	0.69	0.64	0.28
	QSOrder	42	0.95	**0.92**	0.84	0.88	0.77

Best performance metrics are shown in bold.

As can be seen, AAC is the best among all single features, with AUROC of 0.91 and 0.96 in 10-fold cross-validation and independent data test, while the worst was SOCNumber, with AUROC of 0.70 and 0.73. This result should show that SOCNumber has only two dimensions, so this feature cannot afford enough information. Thus, this feature may be used to fuse other features to supplement additional information.

Amino acid composition is only a basic feature and does not burden physicochemical properties. Therefore, we think that there is still a large space for optimization. Previous studies have shown the relationship between bitter peptides and factors such as amino acid hydrophobicity and amino acid position. Some single features with poor performance have rich information that AAC does not have and can improve prediction performance. Therefore, we will study how to optimize the parameters of characteristics in following section.

### 3.2. Fusion feature processing

By fusing the 10 features mentioned above, we will get a 1,337-dimensional fusion feature. In this step, we de-zero the fusion feature. When a column contains only zero, it has no practical effect on the discrimination and is removed. After deleting all zero columns, 1206 features remain, as shown in detail in Table 2.

**TABLE 2:** Features after feature reduction operation.

Feature	Dimension	Dimension after operation
AAC	20	20
TPAAC	21	21
APAAC	22	22
ASDC	400	366
DPC	400	303
DDE	400	400
GAAC	5	5
GDPC	25	25
SOCNumber	2	2
QSOrder	42	42
Total of features	1,337	1,206

### 3.3. Fusion-feature–based results

**FIGURE 2:**
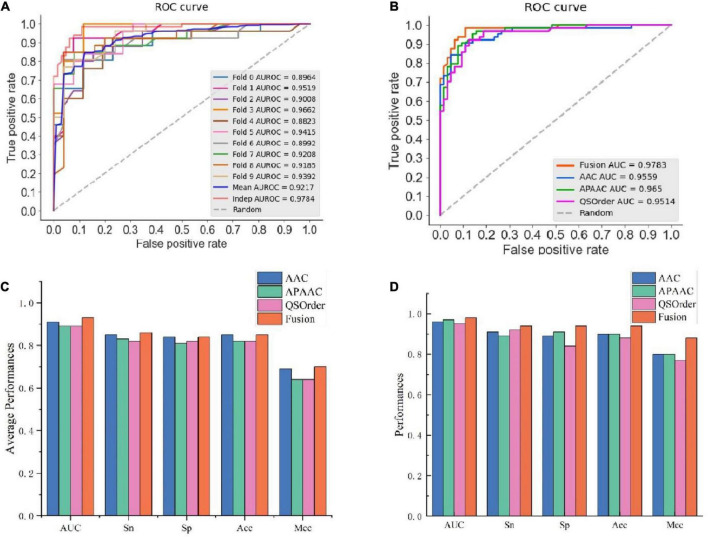
The prediction results using different features. **(A)** AUROC curves of fused features using RF; **(B)** AUROC curves of fusion features and three single-feature on independent data; **(C)** detailed results on training data using 10-fold cross-validation; **(D)** independent data validated results.

In this study, we compared the prediction effect of the fusion features and the three features with the highest independent set validation AUROC value among the above 10 single features. It has been proved that using the RF method to deal with fused features does have more advantages in terms of predictive ability. Table 3 and Figure 2 show the results of 10-fold cross-validation and independent set validation.

**TABLE 3:** Comparison between single-features and fusion feature using RF algorithm.

ML method	Cross-validation	Feature	Dimension	AUROC	Sn	Sp	Acc	Mcc
Random Forest	10-fold cross-validation	AAC	20	0.91	0.85	**0.84**	**0.85**	0.69
		APAAC	22	0.89	0.83	0.81	0.82	0.64
		QSOrder	42	0.89	0.82	0.82	0.82	0.64
		Fusion	1206	**0.93**	**0.86**	**0.84**	**0.85**	**0.70**
	Independent set validation	AAC	20	0.96	0.91	0.89	0.90	0.80
		APAAC	22	0.97	0.89	0.91	0.90	0.80
		QSOrder	42	0.95	0.92	0.84	0.88	0.77
		Fusion	1206	**0.98**	**0.94**	**0.94**	**0.94**	**0.88**

Best performance metrics are shown in bold.

It could be seen that, in 10-fold cross-validation and independent set validation, the prediction performance of fusion features was improved or remained unchanged compared with single feature prediction. That is to say, the fusion features have better predictive ability.

### 3.4. Comparison with other machine learning methods on fusion features

**FIGURE 3:**
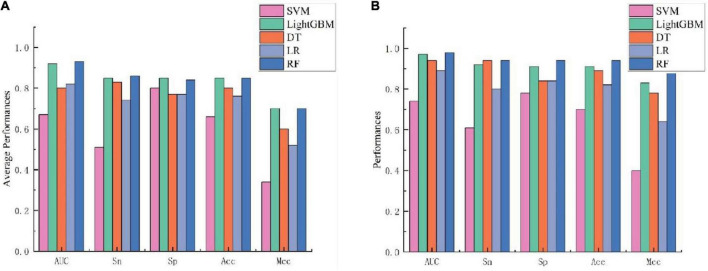
Performance evaluation of different machine learning models using **(A)** 10-fold cross-validation and **(B)** independent testing.

To further validate the prediction model of the RF method for bitter peptides, we compared it with some traditional machine learning methods. Here, Support Vector Machines (SVM), LightGBM, Decision Trees (DT), and Logistic Regression (LR) were selected to build models for comparison. The prediction results of each machine learning method are shown in Table 4 and Figure 3. It can be seen that the RF method is superior to or equal to other machine learning methods in various indicators, and has good learning effect and prediction ability. Therefore, according to the data characteristics provided by us, the RF method shows the best predictive ability.

**TABLE 4:** Comparison of multiple machine learning methods using fusion features.

Cross-validation	Feature	ML method	AUROC	Sn	Sp	Acc	Mcc
10-fold cross-validation	Fusion	SVM	0.67	0.51	0.80	0.66	0.34
	Fusion	LightGBM	0.92	0.85	**0.85**	**0.85**	**0.70**
	Fusion	DT	0.80	0.83	0.77	0.80	0.60
	Fusion	LR	0.82	0.74	0.77	0.76	0.52
	Fusion	RF	**0.93**	**0.86**	0.84	**0.85**	**0.70**
Independent set validation	Fusion	SVM	0.74	0.61	0.78	0.70	0.40
	Fusion	LightGBM	0.97	0.92	0.91	0.91	0.83
	Fusion	DT	0.94	0.94	0.84	0.89	0.78
	Fusion	LR	0.89	0.80	0.84	0.82	0.64
	Fusion	RF	**0.98**	**0.94**	**0.94**	**0.94**	**0.88**

Best performance metrics are shown in bold.

### 3.5. Comparison with existed models

**FIGURE 4:**
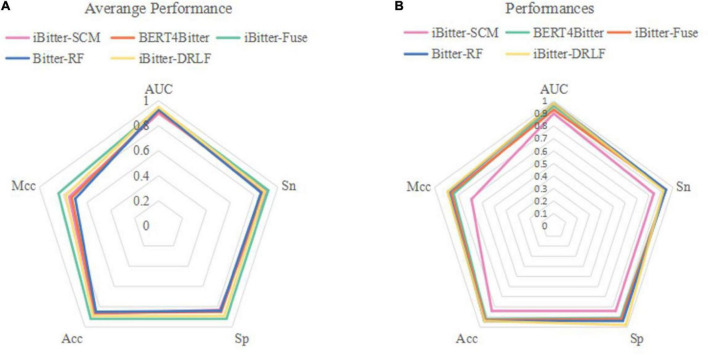
Radar plot for comparing Bitter-RF with other published models using **(A)** 10-fold cross-validation and **(B)** independent testing.

To evaluate the predictive ability of Bitter-RF, we compared it with the existing four sequence-based models. The first model is iBitter-SCM which was constructed based on the dipeptide propensity score, the second model is BERT4Bitter using deep learning method, the third model is iBitter-Fuse by combining fuses features with SVM, and the fourth model was iBitter-DRLF by selecting features through deep learning (22–24, 26). Here, Bitter-RF model used the same bitter peptide and non-bitter peptide sequences as the previous four models. We further extended the types of extracted features on the basis of the third model, and used the RF method for modeling. By referring to relevant literatures, we obtained the performance indicators of the four models. The comparison results have been shown in Table 5 and Figure 4.

**TABLE 5:** Performance comparison of Bitter-RF with the existing methods.

Cross-validation	Classifier	AUROC	Sn	Sp	Acc	Mcc
10-fold cross-validation	iBitter-SCM	0.90	0.91	0.83	0.87	0.75
	BERT4Bitter	0.92	0.87	0.85	0.86	0.73
	iBitter-Fuse	0.94	**0.92**	**0.92**	**0.92**	**0.84**
	iBitter-DRLF	**0.95**	0.89	0.89	0.89	0.78
	Bitter-RF	0.93	0.86	0.84	0.85	0.70
Independent set validation	iBitter-SCM	0.90	0.84	0.84	0.84	0.69
	BERT4Bitter	0.96	**0.94**	0.91	0.92	0.84
	iBitter-Fuse	0.93	**0.94**	0.92	0.93	0.86
	iBitter-DRLF	**0.98**	0.92	**0.98**	**0.94**	**0.89**
	Bitter-RF	**0.98**	**0.94**	0.94	**0.94**	0.88

Best performance metrics are shown in bold.

The performance comparison between Bitter-RF model and the four models showed that the results of Bitter-RF model in 10-fold cross-validation are similar to BERT4Bitter, and slightly lower than iBitter-Fuse. However, the results of Bitter-RF model on independent data are generally better than those of the first three models, and are comparable to those of the fourth model. Bitter-RF model has the same *Sn* index as the previous two generation models, which is superior to the first generation model. The indexes of *Sp*, *ACC* and *MCC* are better than those of the previous three generations. Furthermore, the AUROC of Bitter-RF model is 5% higher than that of iBitter-Fuse. Although the prediction performance of Bitter-RF is close to that of iBitter-DRLF, we used a traditional machine learning method, which consumes less computing resources. To sum up, Bitter-RF model shows stronger prediction performance and better practical application ability.

To our knowledge, we could not find any alternative bitterness classification studies allowing us to assess the intrinsic robustness of the bitter/non-bitter classification and therefore it cannot be excluded that the model may be affected by the inherent bias of training/test set data.

## 4. Conclusion

Compared with other proteins, there is still much room for related research on bitter peptides, and it has shown potential medical benefits. To better study bitter peptides, we developed a novel model Bitter-RF for predicting bitter peptides, which uses information from multiple perspectives, including sequence internal information and physicochemical properties. By comparison, we concluded that fused features could produce better performance than single features, RF is more suitable for bitter peptide prediction, and Bitter-RF has more application advantages than the four published models. Our research further enriches the application of RF method in the field of protein classification. And Bitter-RF model’s better results also show that enrich physical and chemical properties, location information and other characteristics play an important role in the identification of bitter peptides, which can provide biologists with more directions for biological experiments to verify bitter peptides.

However, one may notice that the features were not optimized. In the future, we will use various of feature selection techniques (83–86) to pick out the best features for improving model’s performance.

Based on the proposed method, a free and easy-to-use python package has been built and accessible at GitHub: https://github.com/ZhangYufei01/Bitter-RF.git, which can help scholars to identify bitter peptides.

## Data availability statement

The original contributions presented in this study are included in this article/supplementary material, further inquiries can be directed to the corresponding authors.

## Author contributions

HD, YZ, and K-JD conceived and designed the study. Y-FZ and Y-HW conducted the experiments and implemented the algorithms. Z-FG, X-RP, and JL performed the analysis. Y-FZ, JL, HD, YZ, and K-JD wrote the manuscript. JL, HD, YZ, and K-JD reviewed and edited the manuscript. HD and K-JD supervised the study. All authors contributed to the article and approved the submitted version.
